# HNF4A Regulates the Proliferation and Tumor Formation of Cervical Cancer Cells through the Wnt/*β*-Catenin Pathway

**DOI:** 10.1155/2022/8168988

**Published:** 2022-01-28

**Authors:** Hong-Mei Ma, Qian Zhang, Xue-Mei Yang, Yan Hu, Juan Zhang, Lin Chen, Bin Zhao, Wen-ting Yang, Rui Xu

**Affiliations:** ^1^Department of Reproductive Medicine, The First Affiliated Hospital of Xi'an Jiaotong University, Shaanxi, Xi'an 710061, China; ^2^Department of Internal Medicine One, Shaanxi Provincial Cancer Hospital, College of Medicine, Xi'an Jiaotong University, Shaanxi, Xi'an 710061, China; ^3^Department of Gynecological Oncology, Shaanxi Provincial Cancer Hospital, College of Medicine, Xi'an Jiaotong University, Shaanxi, Xi'an 710061, China; ^4^Department of Pathology, Shaanxi Provincial Cancer Hospital, College of Medicine, Xi'an Jiaotong University, Shaanxi, Xi'an 710061, China; ^5^Department of Pathology, Shaanxi Provincial People's Hospital, Shaanxi, Xi'an 710061, China; ^6^Epidemiology Research Office, Shaanxi Provincial Cancer Hospital, College of Medicine, Xi'an Jiaotong University, Shaanxi, Xi'an 710061, China

## Abstract

Hepatocyte nuclear factor 4 alpha (HNF4A) is a transcriptional factor which plays an important role in the development of the liver, kidney, and intestines. Nevertheless, its role in cervical cancer and the underlying mechanism remain unknown. In this study, both immunohistochemistry and western blotting revealed that the expression of HNF4A was downregulated in cervical cancer. Xenograft assays suggested that HN4A could inhibit tumorigenic potential of cervical cancer in vivo. Functional studies illustrated that HNF4A also inhibited the proliferation and viability of cervical cancer cells in vitro. In addition, FACS analysis implied that HNF4A could induce cell cycle arrest from the G0/G1 phase to S phase. Further studies suggested that HNF4A downregulated the activity of the Wnt/*β*-catenin pathway. Altogether, our data demonstrated that HNF4A inhibited tumor formation and proliferation of cervical cancer cells through suppressing the activity of the Wnt/*β*-catenin pathway.

## 1. Introduction

Cervical cancer is the fourth most common morbidity and mortality cancer in women worldwide in the latest epidemiological survey on cancers and is the second most common killer cancer in women after breast cancer [1]. It is clear that the incidence of cervical cancer is mainly due to papillomavirus infection [2], while increasing evidence suggests that HPV infection is a necessary but not sufficient factor [3, 4]. Thus, other factors may participate in cervical carcinogenesis. Previous research in our laboratory has shown that a set of stem cell-related genes are involved in the pathogenesis of cervical cancer, such as LGR5 [5], OCT4 [6], NANOG [7], and SOX2 [8]. Therefore, the alteration of the gene expression state cannot be ignored.

The Wnt/*β*-catenin signaling pathway plays important roles in various pathophysiological and physiological processes [9–12]. Activation of the Wnt/*β*-catenin signaling pathway has been reported in various cancers [13–17], including cervical cancer [18, 19]. Activation of the Wnt signaling pathway was considered an important process in the transformation of cervical precancerous lesions into cervical cancer [20, 21].

HNF4A is a member of the ligand-dependent nuclear receptor superfamily [22], which can regulate organism development [23], intestinal development [24], and epithelial-mesenchymal transformation [25]. HNF4A not only serves as a transcriptional factor but also can bind to the enhancer of specific genes to regulate its expression [26]. On the one side, HNF4A could serve as a cancer promoter by facilitating the proliferation and invasion of cancer cells. HN4A was upregulated in hepatocellular carcinoma, and its high expression was correlated with poor differentiation of hepatocellular carcinoma cells and vascular invasion [27]. HNF4A was also described as a marker to distinguish metastases of colorectal adenocarcinomas from pulmonary adenocarcinomas in situ [28]. HNF4A was upregulated in intestinal cancer and could eradicate aberrant epithelial cell resistance to ROS production during intestinal tumorigenesis [29]. HNF4A could distinguish primary gastric cancer from breast metastasis [30]. On the other side, HNF4A also plays antitumor roles in several types of cancer. Inhibition of HNF4A resulted in cyclin downregulation, cell cycle arrest, and tumor growth inhibition [31]. In colon cancer, HNF4A suppresses the EMT process by upregulating E-cadherin and downregulating vimentin [32]. HNF4A downregulation was correlated with poor prognosis in renal clear cell carcinoma [33]. Upregulation of HNF4A was also reported in esophageal cancer [34]. HNF4A could promote malignant transformation of paediatric neuroblastoma cells through MMP-2 and MMP-14 [35]. To the best of our knowledge, there is no research on the function of HNF4A in cervical cancer.

In the present study, we demonstrated that HNF4A was downregulated in cervical cancers. Ectopic expression of HNF4A inhibited tumor formation and proliferation of cervical cancer cells both *in vivo* and *in vitro*. Further study revealed that HNF4A arrested cell cycle progression by downregulating the activity of the Wnt/*β*-catenin pathway.

## 2. Materials and Methods

### 2.1. Clinical Specimens

All cancer clinical specimens including 17 normal cervix and 37 cervical cancer tissue samples were obtained from No. 215 Hospital of Shaanxi Nuclear Industry from January 2015 to December 2018 for IHC. All patients did not receive immunotherapy, chemotherapy, or radiotherapy. Eight normal cervices and 8 cervical cancer fresh tissue samples for western blotting were obtained from the First Affiliated Hospital of Xi'an Jiaotong University.

### 2.2. Bioinformatics

The survival analysis was performed with the Kaplan–Meier plotter (http://kmplot.com/analysis/index.php?p=service&cancer=pancancer_rnaseq) using the data in TCGA [36]. There are 304 cervical cancer specimens in TCGA database (complex epithelial neoplasms), including 253 squamous cell neoplasms, 31 adenomas and adenocarcinomas, 17 cystic, mucinous, and serous neoplasms, and 3 complex epithelial neoplasms. The expression of HNF4A in the normal cervix and cervical cancers was obtained from the GEO database (GDS3233 (https://www.ncbi.nlm.nih.gov/sites/GDSbrowser?acc=GDS3233) and GDS3292 (https://www.ncbi.nlm.nih.gov/sites/GDSbrowser?acc=GDS3292)).

### 2.3. Western Blotting

Fresh tissue or adherent cells were firstly washed with PBS 3 times and then lysed with lysis buffer which was added with a certain concentration of cocktail (Roche, Basel, Switzerland) for 1 h on ice. The cell lysates were centrifuged and were quantified by using BCA kits (Thermo Scientific, New York, NY, USA). Equal amounts of protein were subjected to SDS-PAGE gel and then transferred to PVDF membranes (Millipore, Billerica, MA, USA). After blocking with 5% nonfat milk for 1 h, primary antibodies diluted with 5% skimmed milk were incubated with the membrane overnight at 4°C. HRP-conjugated anti-mouse IgG and anti-rabbit IgG (Thermo Scientific, New York, NY, USA) were used to detect the signal of the primary antibodies. The immunoblot bands were visualized with an enhanced chemiluminescence reagent (Millipore, Billerica, MA, USA) on the Tanon system (Tanon, Shanghai, China). The primary antibodies used were as follows: anti-HNF4A (1 : 2000, EPR16885, Abcam, Cambridge, MA, USA), anti-*β*-catenin (1 : 500, sc-7963, Santa Cruz), anti-c-Myc (1 : 1000, 10828-1-AP, Wuhan, China), anti-cyclin D1(1 : 1000, sc-8396, Santa Cruz), and anti-GAPDH (1 : 1000, 10494-1-AP, Wuhan, China).

### 2.4. Immunohistochemistry and Immunocytochemistry

Paraffin-embedded sections were conventionally dewaxed to water. Then, the antigens of paraffin sections were retrieved by using 10 mM citrate buffer (pH 6.0) for 5 min. Adherent cells on slides were fixed with formalin and permeabilized with 0.1% Triton X-100. The paraffin sections or slides were incubated with specific primary antibodies overnight. HRP-conjugated anti-mouse IgG was used to detect the signal of primary antibodies. The expression of specific protein was visualized using the DAB kits (ZSGB-Bio, Beijing, China). The nucleus of cells was visualized with hematoxylin. PBS was used as a negative control. The immunoreactivity score (IRS) was calculated as previously described [18]. The primary antibodies used were as follows: anti-HNF4A (1 : 50, sc-374229, Abcam, Cambridge, MA, USA), anti-Ki67 (1 : 100, sc-23900, Santa Cruz), anti-cyclin D1 (1 : 100, sc-8396, Santa Cruz), and anti-c-Myc (1 : 100, sc-40, Santa Cruz).

### 2.5. Vector Construction and Cell Transfection

The CDS of HNF4A was cloned by PCR and subsequently inserted into pIRES2-AcGFP (Clontech, Mountain View, CA) to construct pIRES2-AcGFP-HNF4A plasmids. The pIRES2-AcGFP plasmids were used as control vectors. The primers used were shown as follows: HNF4A-CDS-F: CCGGAATTCATGCGACTCTCCAAAA, and HNF4A-CDS-R: CGCGGATCCCTAGATAACTTCCTGCTT.

The Lipofectamine 2000 (Invitrogen, Carlsbad, CA, USA) was used as a transfection reagent according to the manufacturer's instructions. The G418 reagent (MCE, New Jersey, CA, USA) was used as selection pressure for stable transfection cell lines.

### 2.6. Cell Culture, Cell Counting Assays, and MTT Assays

The cell culture methods were the same as previously described [37]. For cell counting assays, 2 × 10^4^ cells were seeded into 35 mm dishes, and the cell numbers of each dish were counted after 1, 3, 5, and 7 days. The growth curves were depicted by using Prism software. For MTT assays, cells were seeded into 96-well plates 10^3^ per well, and the absorbance of each well was performed using 3-(4,5-dimethylthiazole-yl)-2,5-diphenyl tetrazolium bromide (MTT, Sigma-Aldrich) at a wavelength of 490 mm. The viability curves were also depicted by using Prism software.

### 2.7. Tumor Xenograft Assay

A total of 5 × 10^5^ HNF4A-modeified cells and their control cells were injected on both sides of the back of nude mice subcutaneously. The volumes of tumors were measured every 3 days. The nude mice were purchased from Charles River (Beijing, China). The nude mice were all 5 weeks old. There were 6 mice in each group. The procedure of this experiment was approved by the Animal Ethics Committee of Xi'an Jiaotong University.

### 2.8. Cell Cycle Analysis

3 × 10^5^ HNF4A-modeified cells and their control cells were seeded into 35 mm dishes. After 24 h, the cells were harvested and washed with PBS for 3 times. The cells were fixed with precooled 75% ethanol overnight. Before fluorescence-activated cell sorting (FACS), the cells were pretreated with PI (propidium iodide, 1 mg/ml, Sigma-Aldrich, St. Louis, MO, USA) and RNaseA (1 mg/ml, Sigma-Aldrich, St. Louis, MO, USA). The flow cytometer used was FACSCalibur (BD Biosciences, San Jose, CA, USA). The data were analyzed by using FlowJo.

### 2.9. Real-Time (RT) Quantitative PCR

The adherent cells were harvested with the RNAiso reagent (Takara, Osaka, Japan). The extraction of RNA was performed according to the manufacturer's manual. The cDNA was reverse transcribed from RNA using the PrimeScript RT reagent Kit (Takara, Osaka, Japan). The cDNA was used as templates, and the SYBR Premix ExTaq II (Takara, Osaka, Japan) was used for real-time quantitative PCR. The procedure of PCR was finished by using the TianLong TL988 System (TianLong, Xi'an, China). The results were analyzed using the MED-TL-4CH software. The primers used were shown as follows: CTNNB1-F: TCTGAGGACAAGCCACAAGATTACA; CTNNB1-R: TGGGCACCAATATCAAGTCCAA; CCND1-F: AAACAGATCATCCGCAAACAC; CCND1-R: GTTGGGGCTCCTCAGGTTC; MYC-F: CCTGGTGCTCCATGAGGAGA; MYC-R: TCCAGCAGAAGGTGATCCAGAC; GAPDH-F: GCACCGTCAAGGCTGAGAAC; and GAPDH-R: TGGTGAAGACGCCAGTGGA.

### 2.10. Statistical Analysis

The statistical analysis was carried out using SPSS 19.0 software (SPSS Inc., Chicago, IL). The data in this article was all shown as means ± standard deviation of the mean (SD). Gene expression in tumor tissues and cells was compared by the unpaired *t*-test, and paired samples were compared by the paired *t*-test. In all tests, *p* < 0.05 is regarded as statistically significant.

## 3. Results and Discussion

### 3.1. HNF4A Is Downregulated in Cervical Cancer

**Figure 1:**
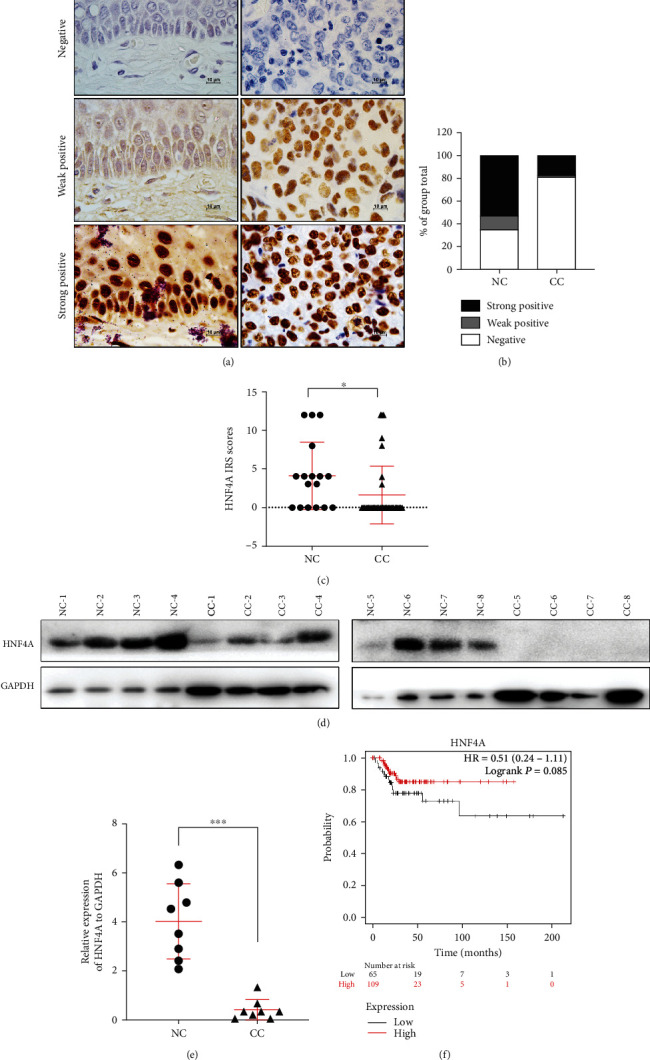
The expression of HNF4A is downregulated in cervical cancer. (a) Immunohistochemical staining of HNF4A in clinical samples, including the normal cervix (NC, *n* = 17) and cervical carcinomas (CC, *n* = 37) (original magnification, 1000x). (b) The immunohistochemical staining intensity was classified into negative, weak positive, and strong positive, and the percentage of each group was shown. (c) The scatter plots showed the IHC scores obtained for the staining of HNF4A in different cervix lesion samples (points represent the IHC score per specimen, and Student's *t*-test is performed). (d) HNF4A expression was detected by western blot in 8 normal cervix samples and 8 cervical carcinoma samples. GAPDH was used as a loading control. (e) The quantitative illustration of the levels of HNF4A protein using densitometry to measure the density of the corresponding bands in (d). Student's *t*-test was carried out. (f) The relationship between relapse-free survival (RFS) probability of CESC patients (*n* = 304) and the expression level of HNF4A in their tumors was shown by the Kaplan–Meier estimator in TCGA database. ^∗^*p* < 0.05, ^∗∗∗^*p* < 0.001.

To explore the function of HNF4A in cervical carcinoma, we conducted immunohistochemistry in the normal cervix (NC) and cervical carcinoma (CC). The results showed that the HNF4A protein was mainly expressed in the nucleus (Figure 1(a)). The positive rate of HNF4A protein in the normal cervix was much higher than that in cervical carcinoma (Table 1 and Figure 1(b), *p* < 0.001). Statistical significance was observed between the two groups (*p* < 0.05). Furthermore, the IHC scores of HNF4A staining were 4.12 ± 4.37 in NC and 1.62 ± 3.74 in CC (Figure 1(c)). We also performed western blotting in eight NC specimens and eight CC specimens (Figure 1(d)). The results showed that the average relative expression of HNF4A in NC was enormously higher than that in CC (Figure 1(e), 4.01 ± 1.535 vs. 0.4169 ± 0.1492, *p* < 0.001). To further understand the expression pattern of HNF4A in cervical cancers, the expression of HNF4A was searched in the GEO database. The relative expression of HNF4A mRNA in GSD3222 was 5.732 ± 3.304 in NC and 4.07 ± 2.03 in CC (Figure S1a, *p* < 0.05). Similarly, the relative expression of HNF4A mRNA in GSD3222 was 9.052 ± 0.4381 in NC and 8.817 ± 0.5171 in CC (Figure S1b, *p* < 0.05). Moreover, the survival analysis in TCGA database (*n* = 304) showed that the patients who had higher expression of HNF4A had a higher relapse-free survival (RFS) probability (Figure 1(f)). Collectively, these data demonstrated that HNF4A was downregulated in cervical cancers and the high expression of HNF4A in cervical cancer may suggest a better prognosis.

### 3.2. HNF4A Inhibits Tumor Formation and Tumor Growth of Cervical Cancer Cells *In Vivo*

**Figure 2:**
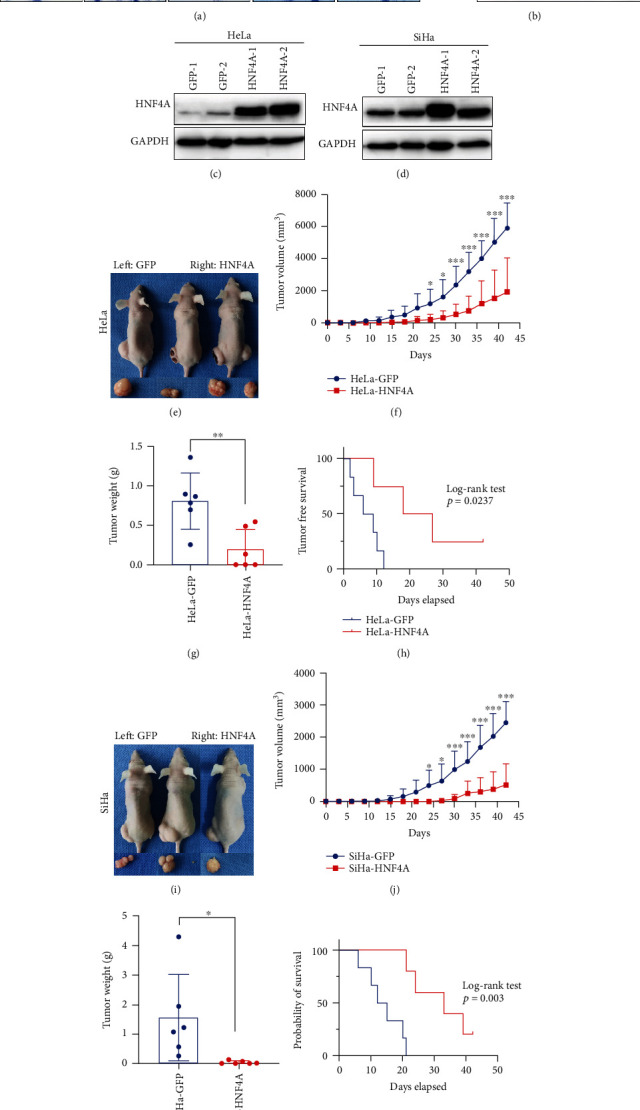
HNF4A inhibits tumor formation and tumor growth of cervical cancer cells in *vivo*. The expression of HNF4A in human cervical cancer cell lines was detected using immunocytochemistry (a) and western blotting (b). Stably transfected HNF4A-modified cervical cancer cells were identified by western blotting (c, d). (e) Xenograft tumor formation of HeLa-GFP and HeLa-HNF4A cells. The tumor growth curve (f), tumor weight (g), and tumor-free survival (h) of HeLa-GFP and HeLa-HNF4A cells, respectively. (i) Xenograft tumor formation of SiHa-GFP and SiHa-HNF4A cells. The tumor growth curve (j), tumor weight (k), and tumor-free survival (l) of SiHa-GFP and SiHa-HNF4A cells, respectively. ^∗^*p* < 0.05, ^∗∗^*p* < 0.01, and ^∗∗∗^*p* < 0.001.

The expression of HNF4A in cervical cancer cells was detected by immunocytochemistry and western blotting (Figures 2(a) and 2(b)). The results showed that HNF4A was expressed in C-33A and SiHa cells but almost not expressed in HeLa, CaSki, and HT-3 cells. Thus, we established stable overexpression of HNF4A in HeLa and SiHa cell lines. The overexpression efficiencies were verified by western blotting (Figures 2(c) and 2(d)).

To illustrate the function of HNF4A *in vivo*, xenograft experiments were carried out in nude mice. HNF4A-modifided cells (HeLa-HNF4A, SiHa-HNF4A) and their control cells (HeLa-GFP, SiHa-GFP) were injected into the backs of the nude mice subcutaneously (Figures 2(e) and 2(i)). The results showed that the tumors developed from HeLa-HNF4A cells grew slower (Figure 2(f), *p* < 0.001) and were lighter than those developed from HeLa-GFP cells (Figure 2(g), 0.1946 ± 0.2991 vs. 0.8092 ± 0.3564, *p* < 0.01). Furthermore, the survival analysis of the tumor-free period in HeLa-HNF4A cells was significantly longer than that in HeLa-GFP cells (Figure 2(h), *p* < 0.05). Similar results were obtained in SiHa-HNF4A and SiHa-GFP cells (Figure 2(j), *p* < 0.001; Figure 2(k), 0.0373 ± 0.0523 vs. 1.559 ± 1.460, *p* < 0.05; Figure 2(i), *p* < 0.01). In conclusion, these results suggested that HNF4A suppressed tumor formation and tumor growth of cervical cancer cells *in vivo*.

### 3.3. HNF4A Inhibits Tumor Formation and Tumor Growth of Cervical Cancer Cells by Inhibiting Cell Proliferation

**Figure 3:**
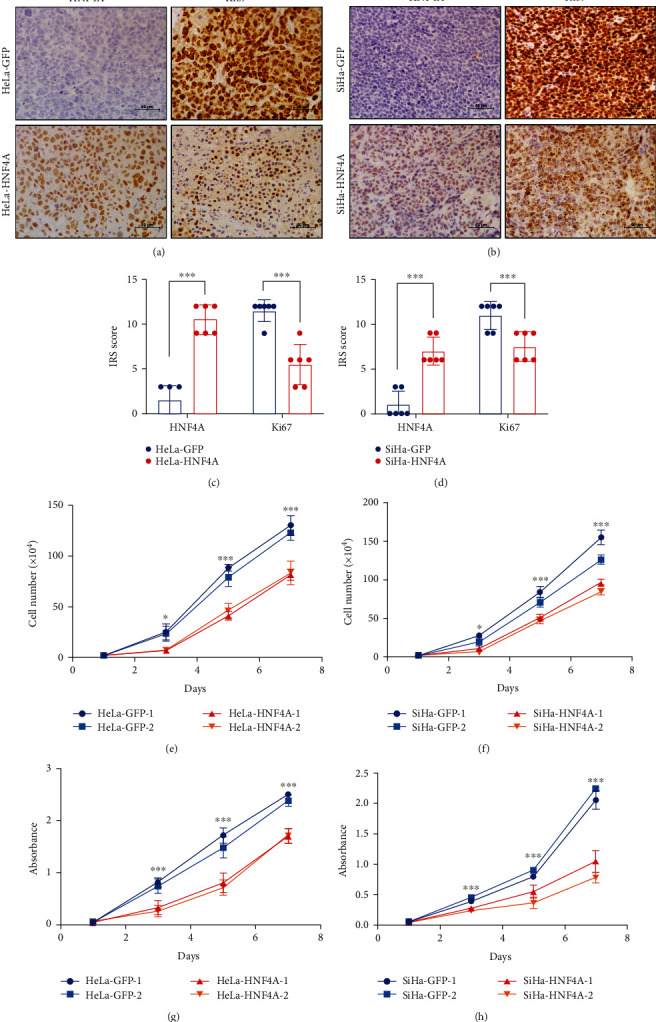
HNF4A inhibits tumor formation and tumor growth of cervical cancer cells by inhibiting cell proliferation. (a, b) Immunohistochemical staining of HNF4A and Ki67 in xenograft tumor tissues derived from HeLa-GFP cells, HeLa-HNF4A cells, and SiHa-GFP and SiHa-HNF4A cells, respectively. (c, d) Immunoreactivity scores of HNF4A and Ki67 in xenograft tumor tissues derived from HeLa-GFP cells, HeLa-HNF4A cells, SiHa-GFP, and SiHa-HNF4A cells. Data were statistically analyzed by Student's *t*-test, and values are shown as mean ± SD. The proliferation was detected using growth curves in HeLa-GFP and HeLa-HNF4A cells (e) and SiHa-GFP and SiHa-HNF4A cells (f). The viability was detected by the MTT assay in HeLa-GFP and HeLa-HNF4A cells (g) and SiHa-GFP and SiHa-HNF4A cells (h). ^∗^*p* < 0.05, ^∗∗∗^*p* < 0.001.

To explore weather HNF4A inhibits tumor formation and tumor growth of cervical cancer cells by inhibiting cell proliferation, the expression of Ki67, which is an important cell proliferation marker, was evaluated in xenografts by IHC (Figures 3(a) and 3(b)). The results suggested that Ki67 staining was weaker in xenografts derived from HeLa-HNF4A cells than in those derived from HeLa-GFP cells (Figure 3(c), HNF4A: 10.5 ± 1.643 vs. 1.5 ± 1.643, *p* < 0.001; Ki67: 5.5 ± 2.258 vs. 11.5 ± 1.225, *p* < 0.001). Similar results were obtained in xenografts derived from SiHa-HNF4A and SiHa-GFP cells (Figure 3(d), HNF4A: 7.1 ± 1.549 vs. 1.1 ± 1.549, *p* < 0.001; Ki67: 7.5 ± 1.634 vs. 11.1 ± 1.549, *p* < 0.001). These results demonstrated that HNF4A inhibits tumor formation and tumor growth of cervical cancer cells by inhibiting cell proliferation.

To further demonstrate the inhibitory function of HNF4A on cell proliferation, the cell growth curve and MTT assays were conducted. It was apparent that after HNF4A overexpression, the cell proliferation ability was severely inhibited (Figure 3(e), *p* < 0.001; Figure 3(f), *p* < 0.001). At the same time, the MTT assays revealed that overexpression of HNF4A significantly suppressed the cell viability of cervical cancer (Figure 3(g), *p* < 0.001; Figure 3(h), *p* < 0.001). These data suggested that HNF4A could inhibit the proliferation and viability of cervical cancer cells *in vitro*. Taken together, these data demonstrated that HNF4A inhibited tumor formation and tumor growth through suppressing proliferation and viability of cervical cancer cells.

### 3.4. HNF4A Inhibits Cell Proliferation through Inducing Cell Cycle Arrest from the G0/G1 Phase to S Phase

**Figure 4:**
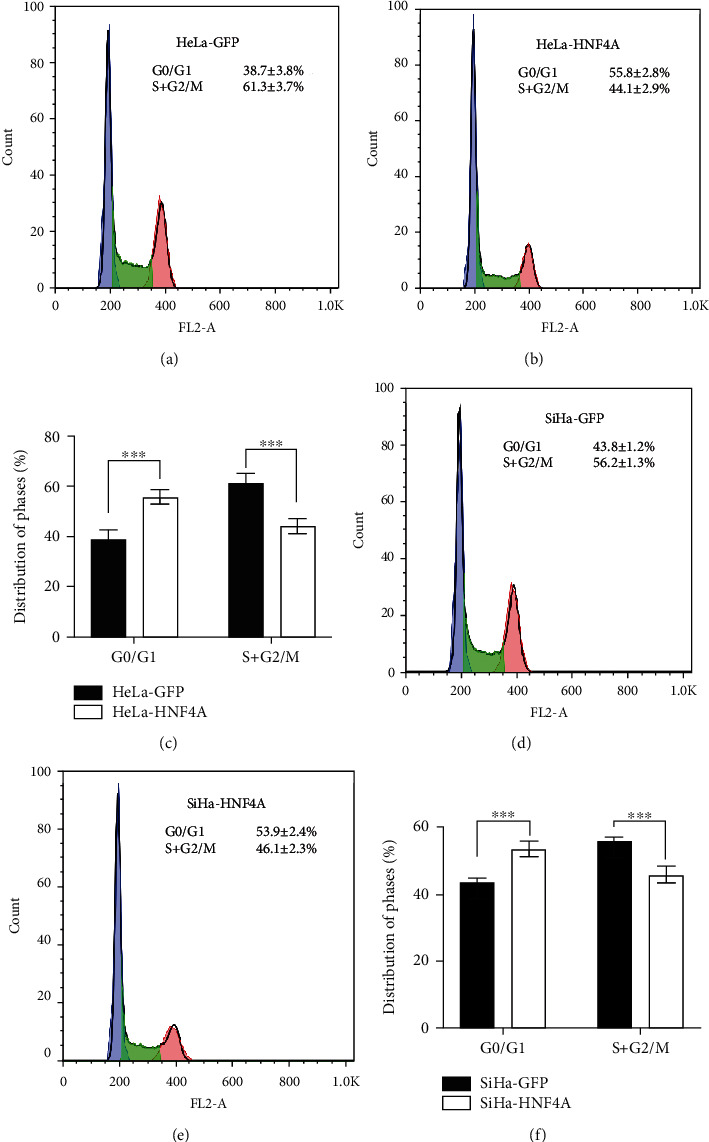
HNF4A inhibited cell proliferation through inducing cell cycle arrest from the G0/G1 phase to S phase. In the flow cytometry figures, the *y*-axis shows the count of effective cells and the *x*-axis shows the DNA content. Each colored area represents the cells of different phases of the cell cycle: blue area refers to the cells in the G0/G1 phase, green area refers to the cells in the S phase, and pink area refers to the cells in the G2/M phase. The cell cycles of HeLa-GFP (a) and HeLa-HNF4A (b) cells were analyzed using flow cytometry, and a quantitative analysis of the cell cycle is shown (c). The cell cycles of SiHa-GFP (d) and SiHa-HNF4A (e) cells and the quantitative analysis (f) are shown. The data were shown as the mean ± SD of three independent experiments. Data were statistically analyzed by Student's *t*-test, and values are shown as mean ± SD. ^∗∗∗^*p* < 0.001.

Cell proliferation is closely related to the cell cycle. The cell cycle is precisely regulated by a set of cell cycle-related proteins, which consist of cyclins, cyclin-dependent kinases (CDKs), and cyclin kinase inhibitors (CKIs), and can be detected by fluorescence-activated cell sorting (FACS). Thus, we performed FACS for cell cycle analysis on HNF4A-modified cells and their control cells. Compared with HeLa-GFP cells, the proportion of HeLa-HNF4A cells in the G0/G1 phase (38.7 ± 3.8% vs. 55.8 ± 2.8%, *p* < 0.001) cells sharply increased, while the proportion of HeLa-HNF4A cells in S and G2/M phases (61.3 ± 3.7% vs. 44.1 ± 2.9%) abruptly increased (Figures 4(a)–4(c)). The same results were observed in SiHa-HNF4A and SiHa-GFP cells (Figures 4(d)–4(f), G0/G1: 43.8 ± 1.2% vs. 53.9 ± 2.4%, *p* < 0.001; S and G2/M: 56.2 ± 1.3% vs. 46.1 ± 2.3%, *p* < 0.001). Collectively, these data illustrated that HNF4A inhibited cell proliferation via arresting cell cycle progression.

### 3.5. HNF4A Arrests Cell Cycle Progression via Downregulating the Activity of the Wnt/*β*-Catenin Pathway

**Figure 5:**
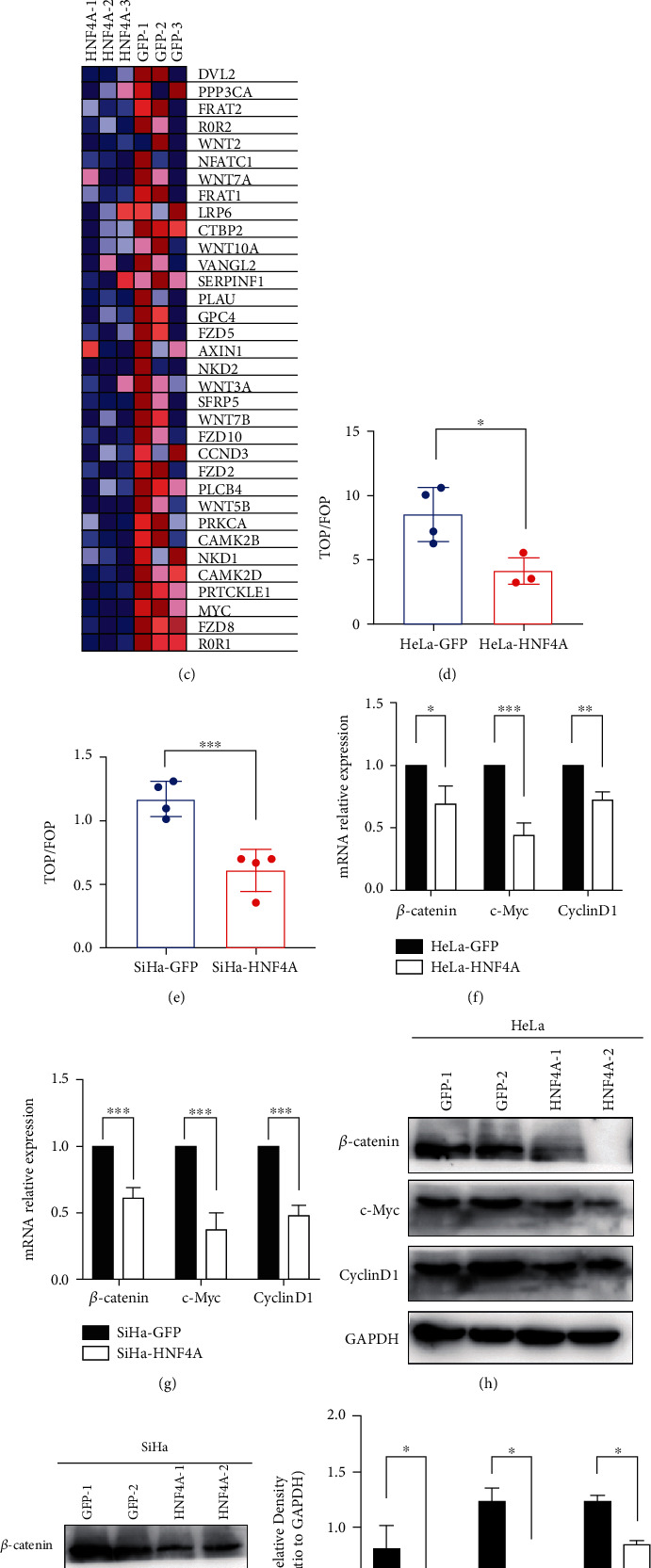
HNF4A downregulated the activity of the Wnt/*β*-catenin pathway. (a) Heatmap of the data from RNA-seq. (b) The result of gene set enrichment analysis. (c) The significantly changed genes in GSEA. (d, e) TOP/FOP-Flash reporter assays were carried out in HNF4A-modified cervical cancer cells. (f, g) Real-time PCR analysis is shown for the mRNA levels of the Wnt/*β*-catenin pathway key genes in HNF4A-modified cervical cancer cells. (h, i) The expression of Wnt/*β*-catenin pathway key proteins in HNF4A-modified cervical cancer cells was determined by western blotting. (j, k) The quantitative analysis of the western blotting in (h) and (i). Data represent mean ± SD of triplicate experiments, and statistical analysis was done by Student's *t*-test. ^∗^*p* < 0.05, ^∗∗^*p* < 0.01, and ^∗∗∗^*p* < 0.001.

To further explore the particular mechanism by which HNF4A regulated the cell cycle, we perform RNA-seq in three SiHa-HNF4A and three SiHa-GFP cells (Figure 5(a)). The RNA-seq results showed that 189 genes were significantly upregulated and 459 genes significantly were downregulated. Next, the gene set enrichment analysis (GSEA) was performed using the data obtained from RNA-seq. The results of GSEA strongly indicated that the Wnt/*β*-catenin pathway was downregulated in HNF4A-overexpressing cells (Figure 5(b) and Supplementary Table 1). The genes that changed significantly are shown in Figure 5(c) (listed in Supplementary Table 2). We conducted the TOP/FOP-Flash luciferase reporter assay, which is always used to measure the activity of the Wnt/*β*-catenin pathway, in HNF4A-overexpressing cells and their control cells. Compared with their control cells, the TOP-Flash luciferase was strongly decreased in HNF4A-overexpressing cells (Figures 5(d) and 5(e)). These data suggested that HNF4A could inhibit the activity of the Wnt/*β*-catenin pathway.

*β*-Catenin is the core molecule of the pathway. At the same time, c-Myc and cyclin D1, which are downstream of the Wnt/*β*-catenin pathway, were involved in cell cycle regulation. To further explore the mechanism underlying, the expression levels of these proteins were measured. At the transcriptional level, the mRNA levels of CTNNB1, MYC, and CCND1 were significantly decreased in HeLa-HNF4A and SiHa-HNF4A cells compared with HeLa-GFP and SiHa-GFP cells (Figures 5(f) and 5(g)). Furthermore, at the protein level, a strong decrease in *β*-catenin, c-Myc, and cyclin D1 was observed in HeLa-HNF4A and SiHa-HNF4A cells (Figures 5(h) and 5(i)). The quantitative analysis of western blotting is shown in Figures 5(j) and 5(k). In conclusion, these data illustrated that the activity of the Wnt/*β*-catenin pathway was downregulated by HNF4A in cervical cancer cells.

### 3.6. HNF4A Suppressed the Wnt/*β*-Catenin Pathway in Mouse Xenografts

**Figure 6:**
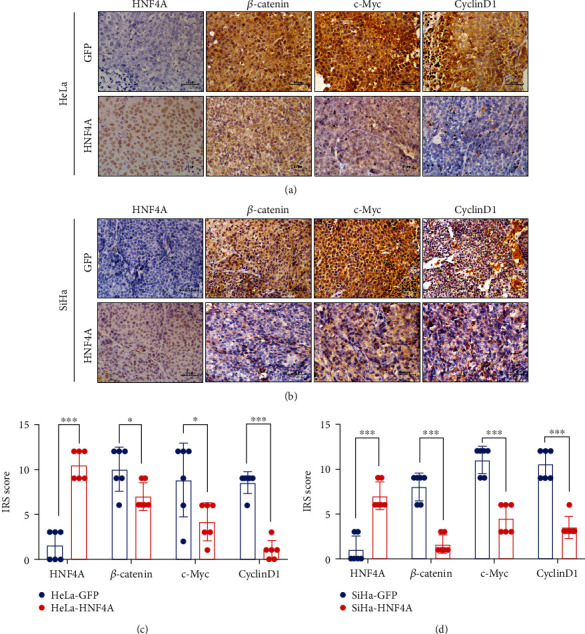
HNF4A suppressed the Wnt/*β*-catenin pathway in a mouse xenograft. (a) Expression of HNF4A, *β*-catenin, c-Myc, and cyclin D1 in tumor xenografts derived from HeLa-GFP cells and HeLa-HNF4A cells. (b) Immunoreactivity scores of HNF4A, *β*-catenin, c-Myc, and cyclin D1 in xenograft tissues derived from HeLa-GFP cells and HeLa-HNF4A cells. (c) Expression of HNF4A, *β*-catenin, c-Myc, and cyclin D1 in xenografts derived from SiHa-GFP and SiHa-HNF4A cells. (d) Immunoreactivity scores of HNF4A, *β*-catenin, c-Myc, and cyclin D1 in xenograft tissues derived from SiHa-GFP cells and SiHa-HNF4A cells. Representative images were shown. Scale bar: 10 *μ*m. Data represent mean ± SD of triplicate experiments, and statistical analysis was done by Student's *t*-test. ^∗^*p* < 0.05, ∗∗∗*p* < 0.001.

To further detect the relationship between HNF4A and Wnt/*β*-catenin pathway, immunohistochemistry was conducted in xenografts derived from HNF4A-modified cells and their control cells with specific antibodies (Figures 6(a) and 6(b)). Compared with xenografts derived from HeLa-GFP cells, the IRS scores of *β*-catenin, c-Myc, and cyclin D1 were strongly decreased in xenografts derived from HeLa-HNF4A cells (Figure 6(c), HNF4A: 10.5 ± 1.643 vs. 1.5 ± 1.643, *p* < 0.001; *β*-catenin: 7.0 ± 1.549 vs. 10.0 ± 2.449, *p* < 0.05; c-Myc: 4.167 ± 2.137 vs. 8.833 ± 4.119, *p* < 0.05; and cyclin D1: 1.0 ± 1.095 vs. 8.5 ± 1.225, *p* < 0.001). Similar results were obtained in xenografts derived from SiHa-GFP and SiHa-HNF4A cells (Figure 6(d), HNF4A: 7.0 ± 1.549 vs. 1.0 ± 1.549, *p* < 0.001; *β*-catenin: 1.667 ± 1.033 vs. 8.0 ± 1.549, *p* < 0.001; c-Myc: 4.50 ± 1.643 vs. 11.0 ± 1.549, *p* < 0.001; and cyclin D1: 3.5 ± 1.225 vs. 10.5 ± 1.643, *p* < 0.001).

Taken together, our results demonstrated that HNF4A inhibited the proliferation and tumor formation of cervical cancer cells through downregulating the activity of the Wnt/*β*-catenin pathway.

## 4. Discussion

HNF4A belongs to the HNF family which plays important roles in regulating the expression of cell-specific genes in many tissues, especially in the liver [38]. HNF4A is not only the main regulator of liver organogenesis but also the tumor suppressor in the liver [39]. The exogenous expression of HNF4A and FOXA3 in hepatoma cells initiated the endogenous expression of a large number of hepatocyte nuclear factors and promoted the transformation of hepatoma cells into hepatocyte-like cells [40]. HNF4A and FOXA2 are important targets which control embryonic hepatoblast differentiation into hepatocytes through the Hippo signaling pathway [26]. Mutations in the HNF4A protein can cause juvenile-onset diabetes mellitus (MODY) [41] and haemophilia [42]. In genome-wide association studies, HNF4A was also described as a susceptibility gene for ulcerative colitis [43]. At the same time, HNF4A is the core factor in the pathogenesis of nonalcoholic fatty liver disease (NAFLD) [44]. Researches about HNF4A in cancer mostly focused on liver cancer, and HNF4A mainly functions as a tumor suppressor in liver cancer [45]. To the best of our knowledge, there is no report on the function of HNF4A in cervical cancer.

In the present study, IHC and western blotting were used to detect the expression level of HNF4A in cervical cancer. The results both revealed that the expression levels of HNF4A protein were low in cervical cancer tissue (Figure 1). Unfortunately, we did not collect enough cervical cancer specimens to measure the mRNA levels of HNF4A. Thus, we turn our attention to the cancer database. The analysis in the GEO database suggested that the mRNA levels of HNF4A were also low in cervical cancer tissue (Figure S1). To further explore the relationship between HNF4A and cervical cancer, the Kaplan–Meier estimator survival analysis in TCGA database revealed that the patients who have higher expression of HNF4A would obtain higher relapse-free survival (RFS) probability. This is the first report that describes the expression of HNF4A in cervical cancer.

Next, HNF4A-modified cell lines were constructed to study the function of HNF4A. Xenograft assays suggested that HNF4A inhibited tumor formation and tumor growth in vivo (Figure 2). Further, MTT and cell counting assays suggested that HNF4A inhibited the proliferation and viability of cervical cancer cells in vitro (Figure 3). Cell proliferation is closely related to the cell cycle which is precisely regulated by a set of cell cycle-related proteins [46]. Previous studies revealed that HNF4A regulated the cell cycle through various approaches. Takashima et al. reported that upregulation of HNF4A combined with two liver-specific transcriptional factors strongly inhibited hepatocellular carcinoma cell proliferation and promoted stem-like cell differentiation into hepatocytes [47]. Li et al. found that HNF4A could downregulate the expression of E2F and thus inhibit the cell cycle process through lncRNAs and miRNAs [48]. Walesky et al. reported that downregulation of HNF4A resulted in the abnormal activation of c-Myc which is an important regulator of the cell cycle [49]. It was also reported that HNF4A suppressed the expression of cyclin D1 in hepatocytes [50]. Cell cycle analysis showed that HNF4A could induce cell cycle arrest from the G0/G1 phase to S phase (Figure 4). Collectively, these data demonstrated that HNF4A could inhibit tumor formation and the proliferation of cervical cancer cells through inducing cell cycle arrest from the G0/G1 phase to S phase. These findings consist in the results of previous studies in hepatocellular carcinoma and strongly imply that HNF4A may induce the cycle arrest through cell cycle-related proteins.

It is well known that the Wnt/*β*-catenin pathway participates in a variety of tumorigenesis processes. Yang et al. found that there was a feedback loop between HNF4A and the Wnt/*β*-catenin pathway during the EMT process in hepatocellular carcinoma [51]. Colletti et al. reported that HNF4A could interact with LEF1, which is a downstream of the Wnt/*β*-catenin pathway, in hepatocellular carcinoma [52]. Another study revealed that the deletion of HNF4A in the liver induced cyclin D1 expression and hepatocyte cell cycle progression [50]. To our knowledge, there is no report about the relationship between HNF4A and the Wnt/*β*-catenin pathway in cervical cancer.

To further detect the mechanism how HNF4A regulated the cell cycle, RNA-seq analysis was performed. The results suggested that the Wnt/*β*-catenin pathway may be involved in this process. TOP/FOP-Flash assays suggested that the Wnt/*β*-catenin pathway was suppressed in HNF4A-overexpressing cells. Subsequent real-time PCR and western blotting showed that the molecules of the Wnt/*β*-catenin pathway were all downregulated at both mRNA and protein levels (Figure 5). The IHC in a mouse tumor xenograft further verified that HNF4A could inhibit the activity of the Wnt/*β*-catenin pathway (Figure 6).

## 5. Conclusions

Altogether, our findings firstly described that HNF4A was downregulated in cervical cancer specimens and HNF4A inhibited tumor formation and proliferation of cervical cancer cells through suppressing the activity of the Wnt/*β*-catenin pathway.

## Figures and Tables

**Table 1:** HHN4A expression levels in different tissue specimens.

Specimens	Total	HNF4A staining		*p*
		Negative, no. (%)	Positive, no. (%)	
Normal	17	6 (35.3)	11 (64.7)	
Carcinoma	37	30 (81.1)	7 (18.9)	<0.001^a^

HNF4A: hepatocyte nuclear factor 4 alpha. The Pearson 2-tailed chi-square test was used to determine the statistical significance of the level of expression of HNF4A in different tissue specimens.

## Data Availability

No data were used to support this study.

## Supplementary Materials

Supplementary 1 Figure S1: the mRNA of HNF4A is downregulated in cervical cancer.

Supplementary 2 Table S1: the GSEA result summary in the WP_WNT_SIGNALING GeneSet.

Supplementary 3 Table S2: detailed rank-ordered gene list for all features in the WP_WNT_SIGNALING dataset.
